# Sudden Intrabulbar Amyloid Increase Simultaneously Disrupts Olfactory Bulb Oscillations and Odor Detection

**DOI:** 10.1155/2019/3424906

**Published:** 2019-08-21

**Authors:** Rebeca Hernández-Soto, Keila Dara Rojas-García, Fernando Peña-Ortega

**Affiliations:** Departamento de Neurobiología del Desarrollo y Neurofisiología, Instituto de Neurobiología, UNAM-Campus Juriquilla, Mexico

## Abstract

There seems to be a correlation between soluble amyloid beta protein (A*β*) accumulation in the main olfactory bulb (OB) and smell deterioration in both Alzheimer's disease (AD) patients and animal models. Moreover, this loss of smell appears to be related to alterations in neural network activity in several olfactory-related circuits, including the OB, as has been observed in anesthetized animals and brain slices. It is possible that there is a correlation between these two pathological phenomena, but a direct and simultaneous evaluation of the acute and direct effect of A*β* on OB activity while animals are actually smelling has not been performed. Thus, here, we tested the effects of acute intrabulbar injection of A*β* at a low dose (200 pmol) on the OB local field potential before and during the presence of a hidden piece of smelly food. Our results show that A*β* decreases the power of OB network activity while impairing the animal's ability to reach the hidden food. We found a strong relationship between the power of the OB oscillations and the correlation between OBs and the olfactory detection test scores. These findings provide a direct link between A*β*-induced OB network dysfunction and smell loss in rodents, which could be extrapolated to AD patients.

## 1. Introduction

Alzheimer's disease (AD) is the most prevalent form of dementia [1]. Aside from cognitive dysfunction, one of the earliest symptoms is hyposmia [2–4]. Recent evidence in animal models has shown that amyloid beta (A*β*) accumulation in the olfactory bulb (OB) and other olfactory-related areas correlates with hyposmia [5–9], especially in the early stages of the AD-like pathology [9–11].

Accumulation of A*β* in a variety of circuits is associated with alterations in their synaptic function and intrinsic neural properties. These alterations are reflected in population activity changes, which seem to underlie cognitive deterioration and other related symptoms in AD [8, 9, 12–16]. Similar alterations in network activity have been observed in the OB upon the acute or prolonged presence of oligomerized A*β* [17–19], which also induces alterations in olfaction [17–21]. Considering that various OB oscillatory activities have been closely linked to olfactory information processing [22–28], it is likely that the A*β*-induced alterations in OB activity contribute to olfactory dysfunction. However, a simultaneous evaluation of both pathological processes is missing. Thus, here, we directly tested this likely pathological relationship by acutely injecting A*β* into the OB while recording its population activity and the animal's ability to locate a source of smell. Our results show that a sudden A*β* application decreases the power of OB network activity and the correlation between both OBs while simultaneously impairing the olfactory function of rats. These findings strongly suggest that A*β* accumulation in the OB and the deterioration of its neural network activity is responsible, at least in part, for the hyposmia observed in AD.

## 2. Materials and Methods

The experimental procedures were approved by the Bioethics Committee of the Institute of Neurobiology at UNAM and were performed in accordance with the guidelines of the Official Mexican Norm for the Use and Care of Laboratory Animals (Norma Oficial Mexicana NOM-062-ZOO-1999) and the Institutional Animal Care and Use Committee Guidebook (NIH publication 80-23, Bethesda, MD, USA, 1996).

### 2.1. Subjects

Experiments were performed using 8-week-old male Wistar rats (300–350 g) obtained from the breeding colony of the animal facility at the Institute of Neurobiology, UNAM. Animals were housed individually in transparent acrylic cages in a temperature-controlled *vivarium* (22 ± 1°C). All animals were kept under a normal 12 h light-12 h dark cycle (lights on at 7:00 a.m.) with food and water *ad libitum*.

### 2.2. Cannula/Electrode Implantation

**Figure 1:**
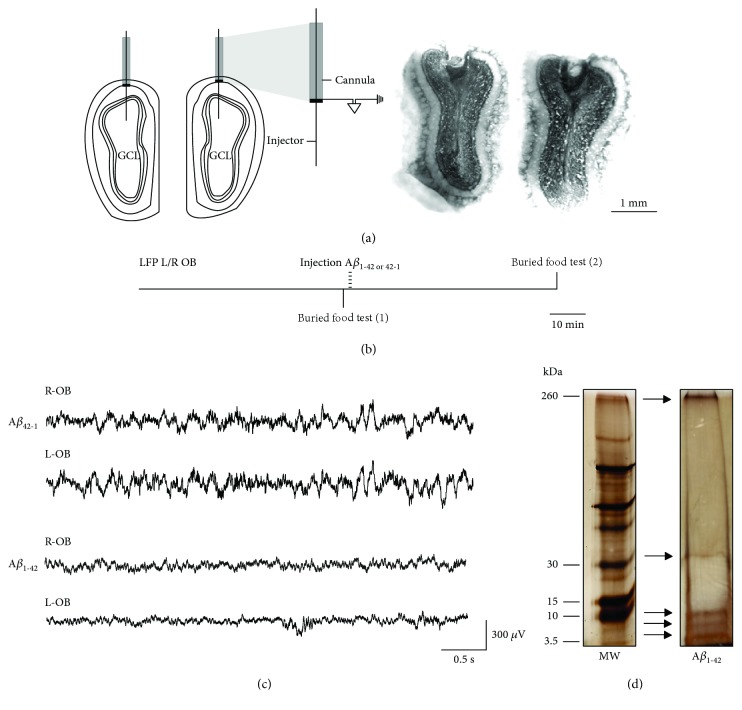
Location of the electrodes and injectors, experimental design, recordings, and composition of the A*β*_1-42_ oligomeric solution. (a) Left: scheme of the OBs and location of a guide cannula in each surface (at the limits between the glomerular and external plexiform layers). The injectors reaching the granular cell layer (GCL) are also represented. One cannula with its corresponding injector is expanded to exemplify that the tip of the cannula is exposed and conductive (in black) for use as a recording electrode. Right: coronal sections of both OBs showing the tracks left by both cannulae and the injectors. (b) Temporal organization (horizontal line) of the local field potential (LFP) recordings of both the left and right (L/R) OBs and the application of two buried food tests: the first (1) just before A*β* administration and the second (2) performed 60 min after A*β* administration. Note that LFP recordings were uninterrupted during the entire experiment. (c) Representative traces of the activity of both OBs 60 min after the administration of either A*β*_1-42_ (upper traces) or its reverse sequence A*β*_42-1_ (lower traces). (d) Silver-stained gel of the electrophoretic pattern of the A*β*_1-42_ oligomerized solution (right lane; monomers, dimers, trimers/tetramers, and heptamers/octamers are the main oligomeric forms) along with a molecular-weight (MW) size marker (left lane).

Animals were anesthetized with sodium pentobarbital (62 mg/kg, i.p.) and then administered atropine sulfate (1 mg/kg, i.p.), saline solution (0.9%, s.c.), and meloxicam (2 mg/kg i.p.). Next, subjects were positioned in a stereotaxic frame (Stoelting Co., IL) for bilateral implantation with stainless steel guide cannulae (23 gauge, 10 mm long) into the superficial layers of the OB (in the boundary between the glomerular and external plexiform layers) at the following coordinates: AP = +7.3, *L* = ±1.3, and *V* = ‐1.1 [29]. Cannulae were used for two purposes: (a) as guides for the microinjectors aiming at the granular cell layer (GCL) and (b) as recording electrodes of the OB field activity. For the latter, cannulae were electrically insulated by varnishing their entire surface except the tips (Figure 1(a)) [30, 31]. The electrodes, including the ground electrode, were attached to male connector pins and inserted into a connector strip (American Phenolic Corp.). Two stainless steel screws were threaded into the cranium over the cerebellum (at the following coordinates: AP = ‐10, *L* = ±3, and *V* = ‐1, [29]) to ground the signal and provide support for the cannulae assembly. The arrangement of cannulae and screws was fixed to the skull with dental acrylic (MDC Dental-NicTone R3V). A stylet was inserted into each cannula and kept in place at all times to avoid obstruction (stylets were only transiently removed for the microinjections and recordings). After the surgical procedure, the animals were allowed to recover for a week before further experimental manipulation.

### 2.3. A*β* Oligomer Preparation

A*β*_1-42_ and its inverse sequence, A*β*_42-1_, were obtained from American Peptide (Sunnyvale, CA). The oligomerization protocol was performed as previously described [14, 19]. Briefly, 1,1,1,3,3,3-hexafluoro-2-propanol (HFIP) was added to solid A*β*_1-42_ at a final peptide concentration of 1 mM and incubated for 60 min at room temperature. HFIP was allowed to evaporate overnight. Then, a 5 mM solution was prepared by adding DMSO. Such solution was then diluted with F12 medium (F12m) to reach a final concentration of 100 *μ*M. This solution was incubated at 5°C for 24 h and then centrifuged at 14,000 rpm for 10 min in the cold. The supernatant containing the A*β* oligomers, monomers, and protofibrils ([14, 19]; Figure 1(d)) was collected and used for the experiments. The inverse sequence A*β*_42-1_ was prepared with the same procedure. The composition of the oligomerized A*β*_1-42_ solution used for this study was corroborated with standard electrophoresis followed by silver staining, which exhibits the presence of monomers, dimers, trimers/tetramers, heptamers/octamers, and large aggregates/protofibrils (Figure 1(d); [14, 19]).

### 2.4. Electrophysiological Recording and Drug Administration

On the day of the experiment, animals were moved to a new cage with the same characteristics as their home (acrylic cage; 24 × 18.5 × 25 cm) and clean sawdust, and then, they were placed in a Faraday cage. There, the OB local field potentials (LFPs) were recorded for 60 min under resting conditions (basal spontaneous activity, baseline) before any behavioral or pharmacological manipulation, and the recordings remained uninterrupted during the entire experiment (Figure 1(b)). The signals were amplified and filtered (high-pass, 0.1 Hz; low-pass, 1.5 kHz) with a differential AC amplifier (A-M Systems, Sequim, WA). The recordings were digitized at 20 kHz and stored in a personal computer with the AxoScope acquisition system from Molecular Devices (Sunnyvale, CA). Then, the buried food tests were performed as described in the next section (Figure 1(b)). After the first olfactory test, either A*β*_1-42_ or its inverse sequence A*β*_42-1_ was bilaterally microinjected. In both cases, 1 *μ*l of a solution containing 100 pmol of A*β* was applied into each OB (200 pmol total). The infusion was made by inserting a microinjector, with a 30-gauge needle (12 mm long), into each guide with the microinjector surpassing it and reaching the GCL (*V* = −3.1). The microinjectors were connected to microsyringes (Hamilton Company, Reno, NV) by polyethylene tubing. The infusion rate, 0.2 *μ*l/min, was controlled by a microinfusion pump (Chemyx Inc., Stafford, TX). After bilateral A*β* infusion, the microinjectors remained in place for 5 min to ensure an adequate A*β* diffusion. After 60 min of postinjection recording under resting conditions (Figure 1(b)), a second olfactory test was performed (Figure 1(b)).

### 2.5. Olfactory Behavioral Test

Olfaction was tested with the “buried food test” [17, 32] while continuously recording the animals. In each test, a piece of chocolate (50 mg; TRIKI-TRAKES®) was randomly placed at one of the four corners, hidden 2 cm under the sawdust. The time that animals took to reach the chocolate (latency) was quantified. The maximum test time allowed was 600 s. The animals were not deprived from food or water at any time, as has been previously reported [33]. The inability or delay to reach the hidden food is interpreted as an alteration in the main OB function [17, 32].

### 2.6. Histological Evaluation

We carried out histological evaluations and used Nissl staining to confirm the injection sites (Figure 1(b); [30]). For this procedure, animals were anesthetized with sodium pentobarbital (70 mg/kg, i.p.) and transcardially perfused with 80 ml of 0.9% saline solution followed by 30 ml of 4% paraformaldehyde (PFA) in 0.1 M phosphate-buffered solution (PBS; pH 7.4). The brains were extracted and maintained in 4% PFA. For sectioning, the OBs were immersed in 0.1 M PBS (pH 7.4), and sagittal slices (40 *μ*m thick) were obtained with a Vibratome (Leica VT1200). Nissl staining was performed as previously described [30].

### 2.7. Data Analysis

**Figure 2:**
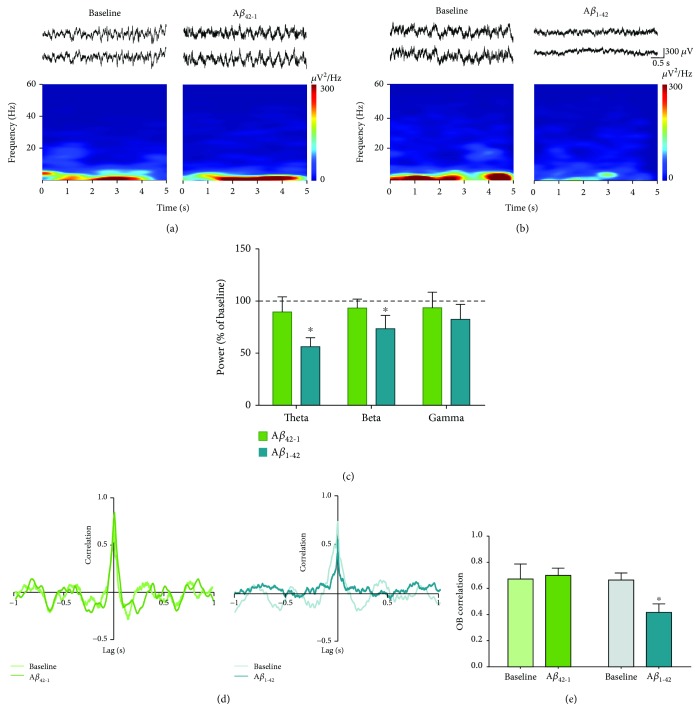
The spontaneous activity of the OB is reduced by the acute administration of A*β*_1-42_. (a) Top: representative recordings of the right (upper trace) and left (lower trace) OBs before (baseline) and 60 min after the administration of the inverse sequence A*β*_42-1_. Bottom: the spectrogram for the recording of one of the OBs. (b) The same as in (a), but for animals given A*β*_1-42_. (c) Quantification of the power (normalized as % of baseline) of the OB activity in the following frequency bands: theta (1-12 Hz), beta (13-35 Hz), and gamma (36-59 Hz), 60 min after the administration of either the inverse sequence A*β*_42-1_ (*n* = 6) or A*β*_1-42_ (*n* = 8). The dotted line represents baseline. (d) Representative cross-correlograms of the activities of both OBs before (baseline) and 60 min after the administration of either the inverse sequence A*β*_42-1_ (left cross-correlograms) or A*β*_1-42_ (right cross-correlograms). (e) Quantification of the peak correlation (at zero lag) of OB activities before and 60 min after the administration of either the inverse sequence A*β*_42-1_ (*n* = 4) or A*β*_1-42_ (*n* = 6). ∗ denotes a significant difference compared to baseline (*p* < 0.05).

**Figure 3:**
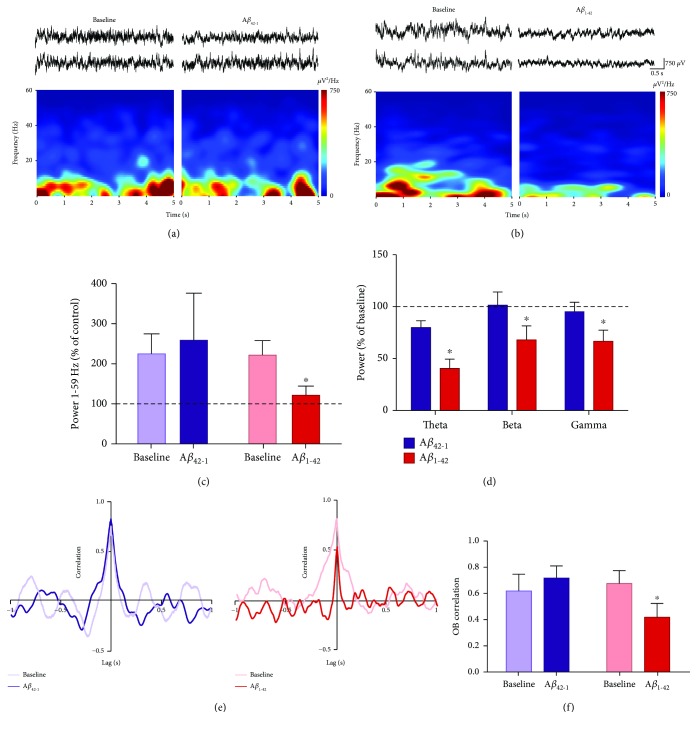
The activity of the OB in the presence of odors is reduced by the acute administration of A*β*_1-42_. (a) Top: representative recordings of the right (upper trace) and left (lower trace) OBs in the presence of odors before (baseline) and 60 min after the administration of the inverse sequence A*β*_42-1_. Bottom: the spectrogram for the recording of one of the OBs. (b) The same as in (a), but for animals given A*β*_1-42_. (c) Quantification of the change in power (normalized as % of control; i.e., period before odor presence) of OB activity in the presence of odors and in broad-band frequency (1-59 Hz) before (baseline) and 60 min after the administration of either the inverse sequence A*β*_42-1_ (*n* = 6) or A*β*_1-42_ (*n* = 8). The dotted line represents control levels. (d) Quantification of the power (normalized as % of baseline) of OB activity in the presence of odors in the following frequency bands: theta (1-12 Hz), beta (13-35 Hz), and gamma (36-59 Hz), 60 min after the administration of either the inverse sequence A*β*_42-1_ (*n* = 6) or A*β*_1-42_ (*n* = 8). The dotted line represents baseline. (e) Representative cross-correlograms of the activities of both OBs in the presence of odors before (baseline) and 60 min after the administration of either the inverse sequence A*β*_42-1_ (left cross-correlograms) or A*β*_1-42_ (right cross-correlograms). (f) Quantification of the peak correlation (at zero lag) of OB activities in the presence of odors before and 60 min after the administration of either the inverse sequence A*β*_42-1_ (*n* = 4) or A*β*_1-42_ (*n* = 5). ∗ denotes a significant difference compared to baseline (*p* < 0.05).

The electrophysiological recordings were analyzed offline by performing the Rapid Fourier Transform Algorithm with a Hamming window in Clampfit 10.6 (Molecular Devices). Segments of 5 s every 10 min were analyzed for spontaneous activity in resting conditions (in the absence of odors) and during the buried food tests. Spectrograms from 1 to 60 Hz were plotted, using NeuroExplorer (5.1), for enhanced visualization of the frequency components of the signals (Figures 2 and 3). The power spectra were segmented in the following frequency bands: theta (1-12 Hz), beta (13-35 Hz), and gamma (36-59 Hz). The power of each individual experiment was normalized to baseline conditions (i.e., activity before A*β* application), arbitrarily set as 100%, for most of the data. To compare the activity power immediately before introducing the odorous food with the activity during the buried food test, the pretest activity power (defined as control) was normalized and set as 100%, only for data presented in Figure 3(c). We evaluated the cross-correlation between the activities of both OBs during resting conditions and in the presence of odors. GraphPad Prism (6.01) was used for statistical analysis. In all cases, data are presented as the mean ± S.E.M. For the comparisons of power quantifications, a Friedman test followed by a Wilcoxon test was performed. The maximum cross-correlation values at zero lag were compared with a Wilcoxon test. The same test was used to compare the latency to find the hidden food. Finally, linear regression analyses were performed to measure the relationship between the latencies to find the hidden food with the maximum correlation values and the power of the OB activity in the different frequency bands.

## 3. Results

### 3.1. Acute A*β* Inhibits OB Spontaneous Network Activity and Decreases the Functional Connectivity between OBs

Histological confirmation of the precise location of cannula tips in both OB superficial layers (at the limits between the glomerular and external plexiform layers) and microinjectors in the GCL (Figure 1(a)) was necessary to include the animals in their respective experimental groups. In one-third of the experiments (5/14), the quality of the recordings (i.e., signal-to-noise ratio) was not optimal in one OB (Figure 1(c)), but the other was properly recorded (also considered minimal for inclusion). In this case, the OB cross-correlation was not performed, but the rest of the quantifications (power and buried tests) were included.

The activity of the OBs, recorded in freely moving rats under resting conditions, exhibited a combination of oscillatory components in a broad frequency range (Figure 1(c)) dominated by slow oscillations, although fast oscillations were also present with less power (Figure 1(c)). The power of this spontaneous network activity in the OBs was reduced by A*β*_1-42_ (Figures 1(c) and 2(b)), but not by its inverse sequence A*β*_42-1_ (Figures 1(c) and 2(a)). The intrabulbar administration of A*β*_1-42_ induced an inhibition of the activity of the OBs, which when quantified after 60 min of its application is reflected in a decrease in theta band power to 56.24 ± 8.55% of baseline (*p* < 0.05, *n* = 8; Figures 2(b) and 2(c)) and beta band power to 73.42 ± 12.79% of baseline (*p* < 0.05, *n* = 8; Figures 2(b) and 2(c)). In contrast, OB activity in the gamma band remained unaltered after A*β*_1-42_ administration (82.40 ± 14.41% of control, *p* = 0.19, *n* = 8; Figures 2(b) and 2(c)). The reduction of OB activity in the presence of A*β*_1-42_ was not observed after the administration of the same dose of its inverse sequence A*β*_42-1_ (Figures 2(a) and 2(c)). In the presence of the inverse sequence A*β*_42-1_, OB activity remained unchanged when quantified in the theta band (89.54 ± 14.49% of control, *p* > 0.05, *n* = 6), beta band (93.36 ± 8.47% of control, *p* > 0.05, *n* = 6), and gamma band (93.47 ± 15.03% of control, *p* > 0.05, *n* = 6; Figures 2(a) and 2(c)). The peak cross-correlation between the spontaneous activities of both OBs at zero lag had a significant reduction compared to baseline (0.41 ± 0.06 vs. 0.66 ± 0.05, *p* < 0.05, *n* = 6) after application of A*β*_1-42_. In contrast, no change in the peak cross-correlation between spontaneous activities of both OBs at zero lag was observed in the presence of the inverse sequence A*β*_42-1_ compared to baseline (0.70 ± 0.05 vs. 0.67 ± 0.11, *p* = 0.4, *n* = 4) (Figures 2(d) and 2(e)).

### 3.2. Acute A*β* Inhibits OB Activity in the Presence of Odors and Decreases the Correlation between OBs

Immediately after evaluating the OB network activity under resting conditions, animals were evaluated in the buried food test, which involved the presence of novel odors in the environment. After the application of either A*β*_1-42_ or its inverse sequence A*β*_42-1_, a second olfactory test was performed (Figure 1(b)). Thus, we also evaluated OB network activity in the presence of novel odors before and after A*β* administration (Figure 1(b)). The activity of the OBs, recorded in the presence of novel odors, exhibited a combination of oscillatory components in a broad frequency range (Figure 3) dominated by slow oscillations, although fast oscillations were also present with low power (Figures 3(a) and 3(b)). OB power increased in the presence of novel odors under baseline conditions (Figure 3(c)). When compared to the period immediately before the presence of novel odors, defined here as “control,” the power of the OB significantly increased to 225.00 ± 50.01% of control in the group subsequently injected with A*β*_1-42_ (*p* < 0.05, *n* = 8) or to 222.00 ± 35.88% of control in the group subsequently injected with inverse sequence A*β*_42-1_ (*p* < 0.05, *n* = 6). This was expected, since both groups shared the same experimental conditions before being injected with one of the two A*β* sequences. The increase in power induced by the presence of novel odors was absent after the application of A*β*_1-42_ (121.60 ± 22.98% of control, *p* = 0.2, *n* = 8) but remained after the application of its inverse sequence A*β*_42-1_ (to 259.00 ± 117.60% of control, *p* < 0.05, *n* = 6) (Figure 3(c)). When examining for the different frequency bands used to assess OB activity under resting conditions, OB activity in the presence of novel odors exhibited a reduction in all frequency bands after A*β*_1-42_ application, but not after the application of its inverse sequence A*β*_42-1_ (Figures 3(a)–3(d)). Compared to the activity of the OBs during the first buried food test before A*β*_1-42_ application (baseline), a reduction in the power of the OB activity for the theta band (to 40.80 ± 9.12% of baseline, *p* < 0.05, *n* = 8; Figures 3(b)–3(d)), beta band (to 68.59 ± 13.60% of baseline, *p* < 0.05, *n* = 8; Figures 3(b)–3(d)), and gamma band (to 67.21 ± 10.82% of baseline, *p* < 0.05, *n* = 8; Figures 3(b)–3(d)) was observed during the second olfactory test performed 60 min after A*β*_1-42_ application. In contrast, rats administered with the inverse sequence A*β*_42-1_ did not show any change in OB power for the theta band (80.53 ± 6.69% of baseline, *p* > 0.05, *n* = 6; Figures 3(a) and 3(d)), beta band (102.30 ± 12.74% of baseline, *p* > 0.05, *n* = 6; Figures 3(a) and 3(d)), and gamma band (96.09 ± 8.99% of baseline, *p* > 0.05, *n* = 6; Figures 3(a) and 3(d)) during the second olfactory test. To determine if the described effects on power are related to changes in the functional connectivity between OBs, we performed a cross-correlation analysis (Figures 3(e) and 3(f)) and found a significant decrease in the peak correlation of OB activity in the presence of novel odors after A*β*_1-42_ administration (0.41 ± 0.10, *p* < 0.05, *n* = 5) during the second buried food test, compared to the first (0.72 ± 0.10). No decrease in peak correlation was found in the second test after the injection of the inverse sequence A*β*_42-1_ (0.68 ± 0.1, *p* < 0.05, *n* = 4), compared to the first test (0.62 ± 0.13) (Figures 3(e) and 3(f)).

### 3.3. Acute A*β* Inhibits Odor Detection, Which is Related to Changes in OB Activity and Correlation

**Figure 4:**
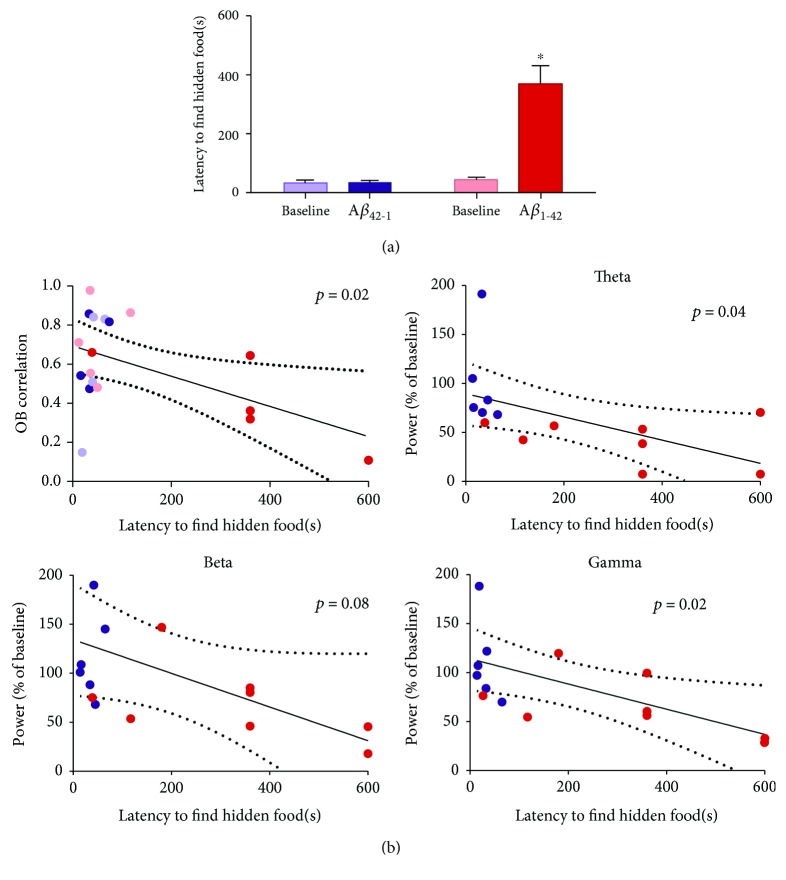
Olfaction is reduced by the acute administration of A*β*_1-42_, which is related to the deterioration of the OB activity and cross-correlation. (a) Quantification of the time to reach a hidden piece of smelly food before (Baseline) and 60 min after the administration of either the inverse sequence A*β*_42-1_ (*n* = 6) or A*β*_1-42_ (*n* = 13). (b) Linear regression analyses to evaluate the relationship between olfactory function, measured as the latency to find a hidden piece of smelly food, and the activity of the OB in the following frequency bands: theta (1-12 Hz; top right), beta (13-35 Hz; bottom left), and gamma (36-59 Hz; bottom right), as well as the correlation between the activities of both OBs (top left). ∗ denotes a significant difference compared to a control test (*p* < 0.05).

To assess the effect of acute administration of A*β*_1-42_ on olfaction, we performed the buried food test before and after its administration (Figures 1(b) and 4(a)). Under baseline conditions, the animals reached the hidden food in 32.83 ± 24.40 s in the group subsequently injected with A*β*_1-42_ (*n* = 13) or 44.54 ± 8.00 s (*n* = 6). This was expected, since both groups shared the same experimental conditions before being injected with one of the two A*β* sequences. The application of A*β*_1-42_ increased the latency to find the hidden food to 369.20 ± 61.76 s (*p* < 0.05, *n* = 13; Figure 4(a)). In contrast, the latency remained unaltered after the application of the inverse sequence A*β*_1-42_ (34.50 ± 19.00 s, *p* = 0.3, *n* = 6; Figure 4(a)). Finally, we also observed that the latency to find the hidden food is related to the level of correlation between OBs (*p* < 0.05; Figure 4(b)) and the power of OB activity during the olfactory test in the theta and gamma bands (*p* < 0.05; Figure 4(b)) but not in the beta band, which only shows a trend of correlation with the olfactory function (*p* = 0.08; Figure 4(b)).

## 4. Discussion

Our results show that a sudden intrabulbar increase in A*β* oligomers induces a reduction in the OB spontaneous network activity and in the correlation between OBs, which are associated with a notorious impairment in olfactory detection. Thus, our findings provide direct evidence of the relationship between A*β*-induced OB activity deterioration and olfactory dysfunction, which has been previously suggested by independent experiments showing disturbances in OB activity and olfaction in transgenic animal models of AD [5, 8, 9, 34, 35], and indirect evidence in AD patients [36]. This pathological relationship was also suggested by the observation of acute A*β*-induced alterations in the electrical activity of the OB *in vitro* [17, 18] and chronic A*β*-induced alterations in the electrical activity of the OB *in vivo* [19] correlating with A*β*-induced olfactory impairment [17, 19], always in independent experiments. The relevance of our observation is that both pathological phenomena were observed simultaneously and their quantifications correlated.

The function of the OB depends largely on the orchestrated activity of its neural components [26, 37, 38], which is expressed through the generation of a variety of oscillatory patterns under resting conditions and in response to odor stimulation [26, 37, 38]. Consequently, alterations in OB neurons affect both the coordinated OB network function and olfactory performance [9, 39–42]. As mentioned, this seemed to be the case for A*β* accumulation in the OB [5, 9].

Here, we corroborated previous findings showing that A*β* oligomers reduce neural network activity in OB slices [17], and *in vivo* [19], this inhibition seems to rely on A*β*-induced alterations in mitral/tufted cell firing [9, 18] and the reduction in lateral inhibition within this circuit [18]. Coincidentally, a reduction in dendrodendritic inhibition has also been observed in an AD transgenic mouse model [9]. Although changes in mitral/tufted excitability and synaptic inhibition are likely candidates for A*β*-induced inhibition of OB neural network activity, the specific effects of A*β* on other OB cell populations (i.e., granular cells) and synaptic interactions (i.e., dendrodendritic excitation) need to be evaluated to determine the exact sources of the inhibitory action of A*β* on OB network activity and, consequently, on olfaction.

Neuronal interactions within the OB originate different oscillatory patterns, including theta rhythm (1-12 Hz), which was the oscillatory activity most affected by a sudden increase of A*β* in our experiments. In the OB, theta activity is mostly driven by rhythmic inputs from the olfactory epithelium produced by the mechanotransduction of the inspiratory air inflow to the nose [25, 43]. We have evidence that local application of A*β* in the OB does not affect the breathing pattern in anesthetized animals (data not shown), excluding the possibility that the effects observed in the OB theta rhythm could be related to changes in breathing, but are more likely the result of A*β* effects on OB circuitry.

The rhythmic activity arriving at the glomeruli in theta frequency recruits local inhibition from juxtaglomerular interneurons innervating mitral/tufted cells, which amplifies theta activity within the OB [23]. Considering that OB activity interneurons seem to be affected by A*β* [9, 18], it is likely that OB theta rhythm is deteriorated by the inhibitory actions of A*β* on juxtaglomerular interneurons or its targets, which is similar to the mechanisms proposed to be involved in A*β*-induced degradation of theta activity in other circuits, including the hippocampus [15, 44, 45]. Here, we found that A*β*-induced deterioration of theta activity closely correlates with olfactory dysfunction, which is similar to the relationship between the A*β*-induced reduction of hippocampal theta activity and memory impairment [17]. Considering that restoration of hippocampal theta rhythm can recover cognitive function [46], it would be interesting to test whether restoration of OB theta rhythm can recover olfaction as well [5].

The other OB rhythm highly affected by A*β* is beta activity. Beta rhythm has been associated with odor discrimination and learning [26, 27, 47] and relies on the centrifugal innervation to the OB and on interbulbar connections [26, 27]. Our finding that A*β* induces beta activity deterioration agrees with the observation by Liu and colleagues (2013) of AD transgenic mice exhibiting a reduction in beta coherence between the OBs [35]. We also found that the correlation between both OBs is reduced after the administration of A*β*. This observation is relevant considering that coherent activity between OBs is required for proper olfactory information processing [47–49]. As mentioned, OB beta rhythm also relies on the centrifugal feedback from the piriform cortex and other forebrain structures [26, 27]. The interaction between the OB and forebrain structures is also relevant for proper olfactory information processing [50–52]. Additionally, our findings indicate that A*β* applied into the OB might affect local centrifugal terminals, as we have shown for hippocampal terminals located in the prefrontal cortex [53].

On the other hand, OB gamma oscillations, which are mildly affected by A*β* administration, are related to a variety of olfactory functions including odor detection, odor perceptual processing, olfactory learning, and discrimination, as well as fine odor discrimination [25, 27, 28, 47, 54]. Considering that OB gamma oscillations mostly rely on the activity of granule interneurons [55–58] and their dendrodendritic interactions with mitral/tufted cells [59, 60], it is likely that A*β*-induced deterioration of synaptic inhibition in the OB [18], which might include the one from granule cells, is the source of A*β*-induced reduction of OB gamma activity. In fact, the OB granule cell layer is a preferential site for A*β* accumulation [5, 7, 20, 61], which is related to the level of olfactory dysfunction [20]. Interestingly, the reduction of A*β* accumulation in the OB by pharmacological means reverses olfactory dysfunction [5, 20].

The OB network suffers a reconfiguration process while transiting from the spontaneous network activity in the absence of odors to the odor-induced neural network activity [62], which involves not only an increase in activity power [22, 26, 37, 43, 63–66] but also changes in cellular elements and synaptic interactions required for the transition from one activity pattern to the other [62]. This reconfiguration process seems to be necessary for proper olfactory information processing [26, 37]. Coincidently, here, we show that A*β* administration affects the change in the OB activity pattern induced by the presence of odors, at least with respect to the increase in overall power. A similar reduced increase in power has been observed in AD transgenic mice [5, 35]. Thus, the A*β*-induced reduction in olfaction not only might be related to the alterations in the generation of the different oscillatory patterns discussed here but may also involve an interference with the reconfiguration of the OB network in response to the presence of odors.

A core finding of this study is that the OB network dysfunction is the main source of A*β*-induced olfactory dysfunction, which opens the possibility of understanding the cellular basis of the hyposmia observed in AD [2, 67–70] and identifying molecular targets to treat it. This is particularly relevant considering that olfactory dysfunction has been closely related to the cognitive deficits in AD [71] and predicts the clinical transition from mild cognitive impairment to AD and mortality in AD patients [68, 72]. Moreover, it is likely that correcting the alterations in the olfactory circuits would improve olfaction in AD patients and contribute to correcting cognitive impairment [73–75].

## 5. Conclusion

Our results provide direct evidence of a close relationship between A*β*-induced OB network activity disruption and olfactory impairment, which indicate that some of the pathological manifestations observed in AD can be originated by disturbances in the activity of a variety of neural networks including the OB. Thus, it is likely that restoring normal neural network activity in the OB could restore olfaction and also be beneficial for the functional recovery of other neural networks closely related to the OB such as the entorhinal cortex or the hippocampus. Consequently, it is probable that the alterations in OB network activity induced by A*β* are responsible for the hyposmia observed in AD and impact other cognitive processes relying on related neural networks.

## Data Availability

Data sets of the current study are available from the corresponding author upon reasonable request.
